# The Application of Negative Pressure Wound Therapy in the Treatment of Chronic Venous Leg Ulceration: Authors Experience

**DOI:** 10.1155/2014/297230

**Published:** 2014-02-18

**Authors:** Marek Kucharzewski, Paweł Mieszczański, Katarzyna Wilemska-Kucharzewska, Jakub Taradaj, Andrzej Kuropatnicki, Zbigniew Śliwiński

**Affiliations:** ^1^Department of Descriptive and Topographic Anatomy, Medical University of Silesia, Jordana 19, 41-808 Zabrze, Poland; ^2^Outpatient Surgery Center No. 2, Specialist Hospital No. 2, Batorego 15, 41-902 Bytom, Poland; ^3^Department of General Surgery, NZOZ District Hospital, Skłodowskiej-Curie 23, 46-200 Kluczbork, Poland; ^4^Department of Physiotherapy Basics, Academy of Physical Education in Katowice, Mikołowska 72, 40-065 Katowice, Poland; ^5^Chair of Euroamerican History and Culture, Pedagogical University of Krakow, Karmelicka 41, 31-128 Krakow, Poland; ^6^Department of Foreign Languages, Medical University of Silesia, Jordana 19, 41-808 Zabrze, Poland; ^7^Institute of Physiotherapy, Faculty of Health Sciences, Jan Kochanowski University, 25-317 Kielce, Poland

## Abstract

The aim of the study was to use negative pressure wound therapy (NPWT) in patients with chronic venous leg ulceration. The authors present their experience in treatment of 15 patients whose average ulceration surface area was 62.6 cm^2^. In 10 patients, the ulcers healed within 6 weeks and in the remaining patients within 20 weeks. Based on the results obtained, the authors imply that NPWT is an effective method in the treatment of chronic venous leg.

## 1. Introduction

Negative pressure wound therapy (NPWT), also known as vacuum assisted closure (VAC), subatmospheric pressure dressing (SPD), vacuum sealing technique (VST), foam suction dressing, sealed surface wound suction (SSS), vacuum pack therapy, and sealing aspirative therapy, is used in the treatment of acute and chronic wounds. The treatment requires a vacuum source to create a continuous or intermittent form of negative pressure inside the wound. Doing so removes fluid and exudates infectious materials to aid in wound healing and closure [1–3].

There are many documented cases of NPWT in wound healing throughout history. In fact, it is one of the oldest methods used in wound treatment and can be traced back to 400 BC when the Greeks practiced cupping using heated copper bowls. Hippocrates and his followers used “collection vessels” whose openings were heated and applied directly over wounds to draw out and collect blood and fluids. Cupping as a vacuum therapy has been used for centuries; however, the technique and design changed as cupping spread west. By the end of the 19th century, Professor August Bier defined the concept of cupping by a method of igniting alcohol within a glass and placing a rubber tube on the skin prior to application of the heated cupping glass. In 1908, Bier's hyperemic treatment method was described and since then vacuum therapy has been used for the treatment of all types of open wounds (traumatic, chronic, and postoperative) as well as for the treatment of infections [4].

In 1907, Dr. E. Klapp first used a suction pump for removal of infectious materials in tuberculosis lesions in patient with advanced tuberculosis. In 1952, the use of NPWT with natural sponge, rubber sponge, foam rubber, cellulose sponge, gauze, cotton, and other filler materials was patented in Germany. The descriptions of more contemporary uses of this method come from the former Soviet Union. In the 1970s, NPWT was used for postsurgical tissue repair and for removal of wound fluids. In 1986, the so-called *Kremlin Papers *started to be published in Soviet medical journals. They describe the use of NPWT for removal of wound exudates from postsurgical wounds. Gauze was applied as the dressing medium, a silicone surgical drain was placed under low continuous wall suction and occlusion with secondary dressings [5]. Vacuum sealing was described in Fleischamnn's work [6, 7]. In 1988, Russian authors published an article in which they explored the use of negative pressure for managing suppurative (pus exuding) wounds. The authors treated 338 patients with abscesses, phlegmons, and purulent wounds. 173 patients were treated by traditional incisive-draining methods and 165 patients were treated by using vacuum therapy by the method proposed by the authors. The advantages of vacuum therapy were shown in the acceleration of reparative processes and in shortening the time of treatment [8]. In 1985, Jeter explored a unique combination of products to deliver negative pressure to the wound bed. She pioneered the use of suction to treat wounds utilizing a gauze dressing and wall suction. In cooperation with Chariker, she drew up a clinical study in which they stated that “their closed suction wound drainage system revolutionized the management of enterocutaneous fistulae complicating ventral abdominal wounds.” In 1989, Chariker et al. developed a technique utilizing standard surgical dressings and wall suction to create a “vacuum” that aided in wound healing. Moist gauze was placed over the wound surface and a flat drain inserted over the gauze and covered with an occlusive dressing. The drain was then connected to a standard hospital wall suction source with continuous pressure set at approximately –60 to –80 mmHg. This method later became known as the “Chariker-Jeter technique” [9].

In 1986, Kostiuchenok et al. showed that application of NPWT in combination with surgical debridement resulted in improved wound healing by reducing considerably the bacterial load within purulent wounds [10]. In the same year, Davydov et al. discovered that vacuum therapy significantly affected the healing process by reducing the bacterial burden and septic complications. It was shown that the use of vacuum therapy shortened healing time, stabilized the immune process, reduced scar tissue formation, and, in consequence, reduced hospital stays [11].

In 1997, Morykwas and Argenta studied the use of suction applied to polyurethane foam in wounds. In their study, subatmospheric pressure was applied through a closed system to an open wound for periods of 48 hours. The subatmospheric pressure was directed at the surface of the wound through an interface between the wound surface and a polyurethane sponge, allowing distribution of the negative pressure and use of either a constant or intermittent mode. In conclusion, the authors stated that the application of controlled subatmospheric pressure creates an environment that promotes wound healing [12, 13]. In 1999, Philbeck Jr. et al. found that “healing time can be as high as 61% faster and 38% less costly with combination treatment utilizing a controlled-suction drain system” [14].

By 2003, NPWT was a commonly accepted therapy. Its use has recently been reviewed and results have been published for a wide range of wound types including diabetes, foot ulcers, surgical wound infections, traumatic wounds, skin graft fixation, pressure ulcers, and leg ulcers. It is thought that NPWT promotes wound healing through multiple actions, including the removal of exudate from the wounds to help establish fluid balance, provision of a moist wound environment, a potential decrease in wound bacterial load, a reduction in edema and third-space fluids, an increase in the blood flow to the wound, and the promotion of white cells and fibroblasts within the wound [15–18].

Literature data concerning application of this method for treatment of venous leg ulceration are scarce; that is why the aim of this paper is to present our own experience in using NPWT for treatment of chronic leg ulcers.

## 2. Materials and Methods

### 2.1. Patients

The study comprised 15 patients (8 women and 7 men) with an age span from 53 to 79 years (mean 62.1 years). The ulcer surface area was from 50.80 cm^2^ to 76.20 cm^2^ (mean 60.71 cm^2^) with persistence time from 60 weeks to 112 weeks (mean 76.3 weeks). In 6 patients, the ulcer was situated on the right leg and in 9 on the left one. Full lower extremity motion was observed in 5 patients and limited motion in 10 patients. The ankle brachial index (ABI) varied from 0.9 to 1.1 (mean 0.98). The body mass index (BMI) varied from 27.8 to 38.2 kg/m^2^ (mean 33.3 kg/m^2^). All patients had been previously treated in dermatological and surgical clinics without success.

After clinical examination, the venous origin of the ulcer was confirmed by means of the venous duplex Doppler sonography and ABI measurement. The patients with previous or active deep vein thrombosis were excluded from the study. The additional exclusion criteria were chronic or critical leg ischemia, contraindications to compression therapy, immobilization in orthesis or plastic cast, paresis related to stroke or paraplegia, chronic cardiac failure with peripheral swelling, and systemic infection. In all the cases, diabetes was also excluded on the basis of laboratory data.

Each patient presented history of the index lesion, treatment, and other significant medical conditions. All patients had been previously treated by their personal physicians by means of elastic bandage compression stocking with wound antiseptic lavage and local application of traditional dressing such as hydrogel and hydrocolloid dressing. However, none of these methods resulted in complete healing of the wound within prerandomization period. Each ulcer was classified according to wound morphology, severity, and location. A systematic description of wound and limb appearance was recorded, including edema, erythema, exudation, granulation, and presence of fibrin or eschar.

### 2.2. Methods

In this study negative pressure wound therapy was provided by the Genadyne A4 system (Genadyne Biotechnologies Inc., Hicksville, NY, USA). The system consists of three components: a negative pressure generating unit with a disposable canister, a pad with evacuation tube, and a reticulated, open cell sterile polyurethane or a dense open-pore polyvinyl alcohol foam dressing cut to fit the wound. The system unit is programmed to deliver controlled negative pressure ranging from 50 to 200 mmHg. NPWT was applied to the ulcer as specified by manufacturer's guidelines, and treatment was continued until ulcer closure, sufficient granulation tissue formation for healing by secondary intention. [2, 19] NPWT dressing changes were performed every 48–72 h, not less than three times per week.

Prior to the treatment, a bacterial swab was taken from each ulcer. During the wound dressing and compression changes, the area of the ulcers was constantly measured. The procedure was as follows. At the outset, homothetic congruent projections of the ulcers were plotted onto transparent foil, after which planimetric measurements of the wounds were taken with the use of digitizer Mutoh Kurta XGT-1218A3 (USA). The area of the ulcer was determined once a week until the wound healed completely. All patients received micronized flavonoid fraction (450 mg diosmin, 50 mg hesperidin), 2 tablets of 500 mg once daily.

## 3. Results

We treated 15 patients (8 women and 7 men) with a mean age of 62.1 years (range 53–79 years). The ulcer surface area was from 50.80 cm^2^ to 76.20 cm^2^ (mean 60.71 cm^2^). The mean ulcer duration prior to the treatment with negative pressure was 76.3 weeks the range of 60–112 weeks (Table 1). The mean treatment time with NPWT was nine weeks. Treatment time for 10 patients was six weeks, and for the remaining five patients, the treatment times were 10, 12, 14, 16, and 20 weeks, respectively (Table 2).

**Figure 1:**
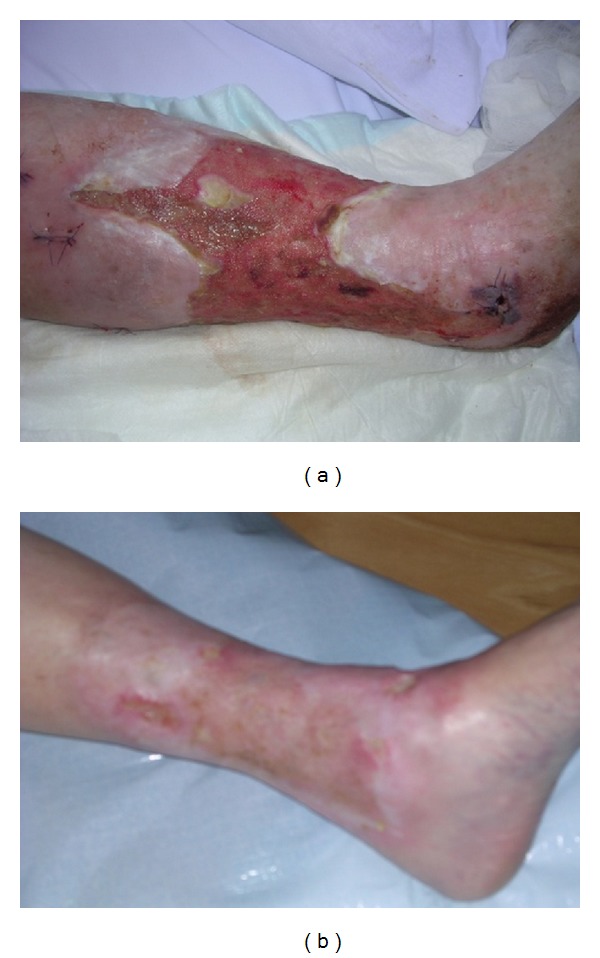
Venous leg ulcer patent number 8 before (a) and after (b) the treatment with NPWT.

We found that in all patients the fibrin on the wound bed was replaced by granulation tissue after one to two weeks (Figures 1(a) and 1(b)). Mean ulcer size was reduced from 15.2 cm^2^ to 13.0 cm^2^–10.6 cm^2^ in the first three weeks of the treatment. In the following weeks, when NPWT was used, mean venous ulcer size got reduced to 4.6 cm^2^–5.7 cm^2^.

## 4. Discussion

Venous leg ulcer is a common ailment, sometimes resulting in disability. Approximately, 2% of the population has a chronic ulcer of the lower limb with female-male ratio 3 : 1. The incidence of venous ulcers increases with age and in the over-65 population it is estimated at the level of 6%. The mean cost of the treatment of leg ulcers in the United States of America is 80 billion dollars per year [20].

Venous leg ulcer is one of the biggest clinical problems in phlebology. Despite epidemiological and pathophysiological knowledge improvement, the number of patients suffering from this complication remains still high stimulating the research focused on the more effective treatment methods. According to the previously performed studies as well as the daily clinical practice, the compression therapy is crucial for the healing of the venous leg ulcers, although the local therapy may also improve the healing rate, if correctly applied to the wound. In this respect, the crucial role of the time strategy and proper wound dressing should also be emphasized including many currently available nonocclusive or occlusive dressings such as hydrogels, hydrocolloids, alginates, or foams [20]. In our study, NPWT was used for the treatment of chronic venous leg ulcers of a surface area greater than 50 cm^2^.

TNWP promotes wound healing through a number of mechanisms. These include edema reduction, increased wound/dermal perfusion, increased granulation tissue stimulation, decreased bacterial loading, and enhanced wound exudates removal [2, 3].

All patients in our study group had had conventional therapy with a mean of 76 weeks before treatment with NPWT. When negative pressure wound therapy was used, complete healing of ulcers was achieved in all patients. Healing time for 10 patients was six weeks, and in the remaining five patients the ulcers healed after 10, 12, 14, 16, and 20 weeks, respectively. In the first three weeks of treatment, the average ulcer surface area was reduced by 24.28%–27.4% and 53%, respectively. In the next weeks of treatment, the ulcer surface area got reduced by 6.7–10%, on average.

Kieser et al. examined 12 patients with chronic resistant venous ulcers. They used NPWT and compression bandaging for 4 weeks. The wounds were monitored for a total of 12 weeks. The authors found statistically significant reductions in ulcer surface area in the first weeks of NPWT therapy [21]. These results are in accordance with ours.

## 5. Conclusions

The results of our study show that negative pressure wound therapy improves the healing process of venous ulcer by decreasing its surface area, which significantly reduces the time of wound treatment.

## Figures and Tables

**Table 1:** Characteristics of the patients.

Patient	Sex	Age (years)	Ulcer surface area (cm^2^)	Ulcer duration (weeks)	Time to completely heal (weeks)
1	Male	53	50.80	60	6
2	Female	60	52.40	62	6
3	Female	64	64.10	70	6
4	Male	58	58.20	68	6
5	Female	59	53.40	64	6
6	Male	60	64.40	72	11
7	Female	66	70.10	96	14
8	Male	61	72.40	100	16
9	Female	72	59.30	68	6
10	Female	68	65.10	76	12
11	Male	55	66.30	80	10
12	Female	63	51.60	70	6
13	Male	54	51.80	68	6
14	Female	79	76.20	112	20
15	Male	59	54.60	78	6

**Table 2:** Patients with healed ulcers according to the duration of treatment.

	Duration of treatment (weeks)																			
	1	2	3	4	5	6	7	8	9	10	11	12	13	14	15	16	17	18	19	20
NPWT						10				1		1		1		1				1
